# Clinical management and outcomes in severe COVID-19: acute respiratory distress syndrome across two waves

**DOI:** 10.1590/1980-220X-REEUSP-2024-0213en

**Published:** 2025-06-20

**Authors:** Vanessa Cláudia Souza Borba, Simone Cristina Soares Brandão, Lúcia Helena de Oliveira Cordeiro, Michele Maria Gonçalves de Godoy, Maria Cristina Falcão Raposo, Romero Carvalho Coimbra Albêlo, Marina Gabinio de Araújo Pontes, Esdras Marques Lins, Emmanuelle Tenório Albuquerque Madruga Godoy

**Affiliations:** 1 Universidade Federal de Pernambuco, Faculdade de Medicina, Recife, PE, Brazil.; 2 Universidade Federal de Pernambuco, Departamento de Clínica Médica, Recife, PE, Brazil.; 3 Universidade Federal de Pernambuco, Programa de Pós-Graduação em Cirurgia, Recife, PE, Brazil.; 4 Universidade Federal de Pernambuco, Departamento de Estatística, Recife, PE, Brazil.

**Keywords:** Respiratory Distress Syndrome, Respiration, Artificial, Critical Care Outcomes, Adrenal Cortex Hormones, COVID-19., Síndrome do Desconforto Respiratório, Respiração Artificial, Resultados de cuidados críticos, Corticosteroides, COVID-19

## Abstract

**Objective:**

Analyze changes in epidemiological and prognostic factors, clinical management and the evolutionary impact of these variables on in-hospital outcomes by comparing the first two waves of Acute Respiratory Distress Syndrome (ARDS) due COVID-19 in a university center in Northeastern Brazil

**Method:**

Patients hospitalized from April 2020 to February 2021 were included in the first wave sample; while the second wave from March to August 2021, according to the rise and fall of cases in Pernambuco. Prospective study where we analyzed the clinical profile, outcomes and treatment in hospitalized patients.

**Results:**

Among 176 patients, 95 were from the first and 81 from the second wave. Mortality was 35,8%, being 47,4% vs. 22,2% (p = 0.001), respectively. Median age was 55 years [IQR:46–58], with no difference between waves. The Sequential Organ Failure Assessment (SOFA) was higher in the first wave, median of 4[IQR: 3;7,7] vs. 3[IQR: 2;5,5], and 5[IQR: 3;8] vs. 3[IQR: 2;7], at 24 and 72 hours, respectively (p = 0.001). Patients in the first wave received more invasive mechanical ventilation (IMV), 68,4% vs. 45,7% (p = 0.002) and hemodialysis, 49,5% vs. 17,7% (p = 0.000), but less non-invasive ventilation (NIV), 8,4% vs. 72,5% (p = 0.000), and corticosteroids, 86,6% vs. 96,6% (p = 0.02). No one was vaccinated in the first wave, while only 7 patients had received a full vaccine in the second wave.

**Conclusion:**

Patients with ARDS had lower mortality, fewer organ dysfunctions and less need for IMV and hemodialysis, with greater use of NIV and corticosteroids in the second wave.

## RESUMO

**Objetivo:**

Analisar as mudanças nos fatores epidemiológicos e prognósticos, no manejo clínico e no impacto evolutivo dessas variáveis nos desfechos hospitalares, comparando as duas primeiras ondas da Síndrome do Desconforto Respiratório Agudo (SDRA) devido à COVID-19 em um centro universitário no Nordeste do Brasil.

**Métodos:**

Os pacientes hospitalizados de abril de 2020 a fevereiro de 2021 foram incluídos na amostra da primeira onda; enquanto a segunda onda, de março a agosto de 2021, de acordo com o aumento e a queda de casos em Pernambuco. Estudo prospectivo em que analisamos o perfil clínico, os desfechos e o tratamento em pacientes hospitalizados.

**Resultados:**

Entre 176 pacientes, 95 eram da primeira onda e 81 da segunda onda. A mortalidade foi de 35,8%, sendo 47,4% vs. 22,2% (p = 0,001), respectivamente. A idade média foi de 55 anos [IQR: 46–58], sem diferença entre as ondas. A Avaliação Sequencial de Falência de Órgãos (SOFA) foi maior na primeira onda, com mediana de 4 [IQR: 3;7,7] vs. 3 [IQR: 2;5,5] e 5 [IQR: 3;8] vs. 3 [IQR: 2;7], em 24 e 72 horas, respectivamente (p = 0,001). Os pacientes da primeira onda receberam mais ventilação mecânica invasiva (VMI), 68,4% vs. 45,7% (p = 0,002) e hemodiálise, 49,5% vs. 17,7% (p = 0,000), mas menos ventilação não invasiva (VNI), 8,4% vs. 72,5% (p = 0,000) e corticosteroides, 86,6% vs. 96,6% (p = 0,02). Ninguém foi vacinado na primeira onda, enquanto apenas 7 pacientes haviam recebido vacina completa na segunda onda.

**Conclusões:**

Os pacientes com SDRA apresentaram menor mortalidade, menos disfunções orgânicas e menor necessidade de VMI e hemodiálise, com maior uso de VNI e corticosteroides na segunda onda.

## INTRODUCTION

The acute phase of severe acute respiratory distress syndrome (ARDS) is characterized by the sudden onset of respiratory failure refractory to oxygen supplementation via an oxygen catheter, non-rebreathing mask and/or non-invasive ventilation (NIV), which may progress to the need for invasive mechanical ventilation (IMV). In particular, deterioration after the first week of adequate treatment indicates a worse prognosis. In a meta-analysis of 25 studies including 4881 patients with severe coronavirus disease-2019 (COVID-19) and non-severe COVID-19, the prevalence of ARDS, renal dysfunction and shock were more common in severe COVID-19 and the mortality of severe COVID-19 was around 30%^(1)^.

If a patient with ARDS develops the need for mechanical ventilation, respiratory monitoring, sedation management, and the use of neuromuscular blocking agents are extremely important, in addition to early therapeutic interventions such as the alveolar recruitment maneuver and the prone maneuver^(2)^. Preventing ventilator-induced lung injury is one of the greatest challenges in the management of ARDS due to the diverse phenotypes of the disease. Understanding how these respiratory interventions were performed is essential^(3)^.

The severity of the disease and the treatments have a direct impact on the sequelae after the acute phase of ARDS. Several groups that have studied quality of life, functionality, and long-term mortality after ARDS have observed several physical, cognitive, and emotional sequelae that fall under the umbrella of Post-Intensive Care Syndrome^(4)^.

Brazil is the third most affected country in the world by COVID-19, with approximately 36 million cases and nearly 700 thousand deaths, with 3.3% occurring in the state of Pernambuco^(5,6)^.

Following the large-scale diagnosis of severe acute respiratory syndrome coronavirus 2 (SARS-CoV-2) in several countries, a complex scenario of infection waves was observed due to the viral mutability, the relaxation of non-pharmaceutical interventions, combined with the low level of acquired immunity^(7)^. Factors related to the virus, the host, and pharmacologic and non-pharmacologic interventions have changed over time. In addition, it is not clear how much this has affected in-hospital outcomes or how much the improvement in clinical management of patients has contributed to the reduction in mortality.

It is essential to know the evolution and behavior of ARDS due to COVID-19 and which factors or interventions may have positively or negatively influenced in-hospital outcomes in each region of the world in order to develop a more effective care and prevention plan in case of a new wave of the disease. Wave behavior has been quite variable around the world. In a study in Saudi Arabia, a reduction in symptoms and mortality was observed in the second wave, while in India, mortality was higher in the second wave^(8,9)^.

There are several retrospective studies in Brazil that outline the epidemiologic and clinical profile and results of a single wave, but there is no prospective study that comparatively analyzes the waves, taking into account therapeutic interventions and their impact on the evolution of the disease in Brazil^(10, 11, 12)^. Therefore, the aim of this study was to analyze the changes in epidemiologic and prognostic factors, clinical management and the evolutionary impact of these variables on in-hospital outcomes, comparing the first two waves in a university center in Northeastern Brazil.

All these lessons learned from the COVID-19 pandemic, especially between the first and second waves, have a positive impact on the way patients with ARDS are managed in order to optimize treatment and reduce mortality and morbidity.

## Method

### Study Design and Population

This is a prospective and observational study. We recruited 241 consecutive patients older than 18 years, admitted to the Intensive Care Unit (ICU) of the Hospital das Clínicas of the Federal University of Pernambuco (EBSERH/HC-UFPE) with a respiratory disease.

Patients hospitalized from April 18, 2020 to February 28, 2021 were included in the first wave sample; while the second wave included patients from March 1 to August 6, 2021, according to the rise and fall of cases in Pernambuco cataloged in the epidemiological bulletins provided by the Center for Strategic Information on Health Surveillance in Pernambuco^(6)^.

There were 65 patients excluded from a total of 241 patients; 55 due to negative COVID-19 real-time reverse transcription-polymerase chain reaction (RT-PCR) test using a nasal swab, six due to lack of RT-PCR test, two patients with advanced terminal illness in palliative care, and two patients due to insufficient data in the medical record. Our sample consisted of 176 patients, 95 in the first wave period and 81 in the second wave period.

### Data Collection

Demographic data were collected (age in years and sex – male/female), length of ICU and hospital stay in days, admission vital signs [heart rate (HR in beats per minute - bpm), respiratory rate (RR in breaths per minute – bpm), systolic blood pressure (SBP) and mean arterial pressure (MAP) in mmHg], Type and number of comorbidities (obesity, hypertension, type 2 diabetes mellitus, chronic obstructive pulmonary disease, asthma, other lung disease, chronic kidney disease, dyslipidemia, previous heart disease), body mass index (BMI in kg/m^(13)^, vaccination status for COVID-19, in-hospital interventions (use and mode of oxygen delivery, use of non-invasive ventilation (NIV), use and number of days of invasive mechanical ventilation (IMV), tracheostomy, use of vasoactive drugs, need for hemodialysis, performance of prone maneuver (yes or no) or alveolar recruitment maneuver (yes or no), use of corticosteroids (yes or no), use of immunosuppressants (yes or no)), use of corticosteroids (type and cumulative dose in milligram equivalents of dexamethasone), use of heparin (type and dose used during the first seven days after admission), use of other medications (sedatives, neuromuscular blocking agents, oseltamivir, ivermectin, and antibiotics), and mortality. The Simplified Acute Physiology Score III (SAPS 3) prognostic scores were calculated at admission, and the Sequential Organ Failure Assessment (SOFA) scores were calculated at admission (24 hours) and 72 hours after ICU admission to measure organ dysfunction (renal, neurological, pulmonary, circulatory, hepatic, or coagulation)^(14,15)^.

For critically ill patients unable to be weighed and measured by traditional methods, height and weight were estimated using the Chumlea formula, which takes into account gender, knee height, calf and arm circumferences, and subscapular skinfold size^(16)^. All of the above variables were collected from medical records and updated daily.

The institutional protocol defined the heparin dose as a prophylactic dose to prevent venous thromboembolism of 40 mg enoxaparin per day or 5,000 UI of unfractionated heparin 2 to 3 times per day, both administered subcutaneously. The therapeutic dose for venous thromboembolism as 1mg/kg enoxaparin every 12 hours or 250 UI/kg unfractionated heparin every 12 hours, while the intermediate dose would be some dose between these two doses.

### Ethics

This project was approved by the Research Ethics Committee of the Hospital das Clínicas of the Federal University of Pernambuco, through Opinion No. 4.165.300, on July 21, 2020. The study participants authorized their participation by signing the informed consent form, or their family members authorized it remotely if they were unable to authorize it for clinical reasons.

### Statistical Analysis

Descriptive statistical analysis was used, including the calculation of arithmetic means, medians, standard deviations, and percentiles for quantitative variables (discrete or continuous) and frequency and percentage analysis for categorical data.

The Mann-Whitney test was used to compare means/medians, and Pearson’s chi-squared test of independence was used to examine associations.

Comparative analyses between waves were performed using possible explanatory variables with a significance level (p-value) lower than 0.05. Statistical analyses were performed using SPSS Statistics for Windows, version 20.0.

## RESULTS

A total of 98 of the 176 patients selected were male (55.7%) (p = 0.235). The median age was 55.5 years [IQR] [46;58], with no statistical difference in gender and age between the two waves (p = 0.10). ICU length of stay was similar in both waves, with a median of 7 days in both IQR (3;18) vs. (3;13.5) for the first and second waves, respectively (p = 0.335). However, the length of hospital stay was longer in the second wave, 12 IQR [6; 27] vs. 17 days [10; 32.5] (p = 0.037).

The median number of comorbidities was not statistically different between Wave 1 and Wave 2 (p = 0.140), but there was a trend towards a higher number of comorbidities (≥2 comorbidities) in Wave 1, with diabetes being the most common. The number of patients with chronic kidney disease (creatinine clearance <30 mL/min) was statistically higher in wave 1 (Table 1).

**Table 1:** Characterization of clinical comorbidities in study patients hospitalized with severe COVID-19 in an intensive care unit of a university hospital during the 1st and 2nd waves of the pandemic – Recife, PE, Brazil, 2020-2021.

Comorbidities	Total sample N = 176 n (%)	1st wave N = 95 n (%)	2nd wave N = 81 n (%)	p-value
Obesity (BMI > 30 kg/m^2^)	78 (48.8)	34 (35.8)	44 (54.3)	0.153(*)
Nutritional classification			
<30	82 (51.3)	45 (57.0)	37 (45.7)	0.507(*)
30–35	44 (27.5)	18 (22.8)	26 (32.1)	
35–40	19 (11.9)	9 (11.4)	10 (12.3)	
> 40	15 (9.4)	7 (8.9)	8 (9.9)	
Hypertension	99 (56.3)	53 (55.8)	46 (56.8)	0.894(*)
Diabetes Mellitus Type 2	51 (29.0)	33 (34.7)	18 (22.2)	0.068(*)
Chronic obstructive pulmonary disease	7 (4)	4 (4.2)	3 (3.7)	0.864(*)
Asthma	10 (5.7)	3 (3.2)	7 (8.6)	0.117(*)
Other chronic lung disease	5 (2.8)	4 (4.2)	1 (1.2)	0.236(*)
Chronic kidney disease	25(14.2)	20 (21.1)	5 (6.2)	0.005(*)
Dyslipidemia	3 (1.7)	2 (2.1)	1 (1.2)	0.656(*)
Previous heart disease	24 (13.6)	15 (15.8)	9 (11.1)	0.367(*)
Quantity of comorbidities			
0	55 (31.3)	29 (30.5)	26 (32.1)	
1	53 (30.1)	23 (24.2)	30 (37.0)	
2	40 (22.7)	24 (25.3)	16 (19.8)	
3	21 (11.9)	13 (13.7)	8 (9.9)	
4	7 (4.0)	6(6.3)	1 (1.2)	
Median (P_25_; P_75_)	1,0 (0,0; 2,0)	1,0(0,0; 2,0)	1,0 (0,0; 2,0)	0.140(**)
Quantity of comorbidities			
Up to 1	108 (66.4)	52 (54.7)	56 (69.1)	0.051(*)
≥2	68 (36.6)	43 (45.3)	25 (30.9)	

(*)p-value of Pearson’s Chi-squared test of independence;

(**)p-value of the Mann-Whitney test. Abbreviation: BMI = Body Mass Index.

**Figure 1:**
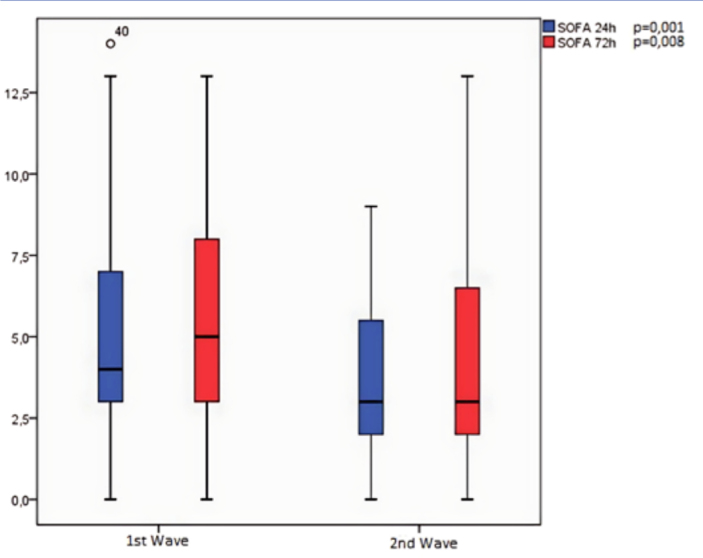
Comparative analysis of 24-hour and 72-hour SOFA scores between the 1st and 2nd waves of COVID-19 in a public teaching hospital – Recife, PE, Brazil, 2020-2021. *p-value of the Mann-Whitney test.

Abbreviation: SOFA = Sequential Organ Failure Assessment.

In the first wave, 9 (5%) patients had SBP <90 mmHg on admission versus 1 (1.2%) in the second wave (p = 0.03). The mean HR was also statistically higher in the first wave (99 bpm × 92 bpm, 1st vs. 2nd wave, p = 0.021). There was no difference in the number of patients with MAP < 65 mmHg (0 vs. 5 ptes, 1st vs. 2nd wave, p = 0.136) and mean respiratory rate (29 bpm vs. 26 bpm, 1st vs. 2nd wave, p = 0.284). The 24-hour and 72-hour SOFA scores were lower at wave 2 (p = 0.001) (Figure 1). However, the SAPS3 score did not show a statistical difference (median 14.5 (8;34.5) in wave 1 vs. 15.9 (7.2;20.5) in wave 2, p = 0.326).

**Figure 2:**
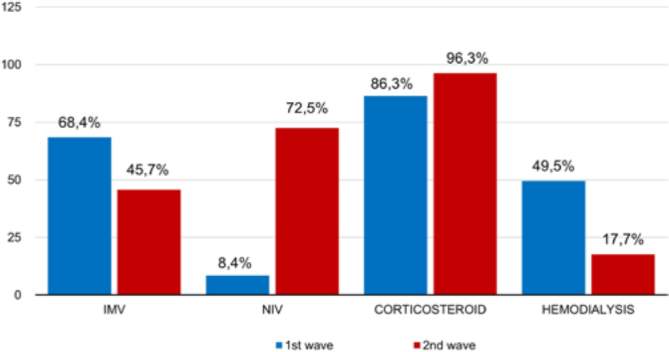
Comparative analysis of the percentage of patients requiring invasive mechanical ventilation, non-invasive ventilation, corticosteroids and hemodialysis between the 1st and 2nd waves of severe COVID-19 in a public teaching hospital – Recife, PE, Brazil, 2020-2021.

Abbreviation: Invasive Mechanical Ventilation (IMV), Non-Invasive Ventilation (NIV).

More oxygen was used in the form of an oxygen catheter (63.2% vs. 40%, p = 0.002) and non-rebreather mask (94.7% vs. 41.3%, p = 0.000) in wave 1, as more patients were also placed on IMV (68.4% vs. 45.7%, p = 0.002) and underwent hemodialysis (49.5% vs. 17.7%, p = 0.000). However, fewer patients received corticosteroids (86.3% vs. 96.3%, p = 0.022) and NIV (8.4% vs. 72.5%, p = 0.000) in wave 1 (Figure 2).

The median number of days on mechanical ventilation was 11.5 [IQR] [7;19] and there was no statistically significant difference between waves. Approximately 13.7% (n = 13) of patients had a tracheostomy in the first wave and 20% (n = 16) in the second wave, but without statistical significance (p = 0.263). The use of vasoactive medications was similar between waves (45.3% vs. 42.5%; p = 0.714), as was the percentage of patients undergoing alveolar recruitment and prone maneuvers (52.6% vs. 55% (p = 0.754); and 72.6% vs. 65.6% (p = 0.12), respectively). The median cumulative equivalent dose of dexamethasone was 100 mg [IQR] [13;175] and was not statistically different between waves (p = 0.178). There was greater use of oseltamivir, ivermectin and sedation in the first wave (Table 2).

**Table 2:** Characterization of medication use in study patients hospitalized with severe COVID-19 in the intensive care unit of a University Hospital during the 1st and 2nd waves of the pandemic – Recife, PE, Brazil, 2020-2021.

Variable	Total sample N = 176 n (%)	1st wave N = 95 n (%)	2nd wave N = 81 n (%)	p-value
Medications				
Antibiotics	169 (96)	93 (97.9)	76 (93.8)	0.169(*)
Oseltamivir	55 (31.3)	54 (56.8)	1 (1.3)	0.000(*)
Ivermectin	51 (29)	43 (45.3)	8 (10)	0.000(*)
Sedation	95 (54)	60 (63.2)	35 (43.8)	0.010(*)
Neuromuscular blocker	78 (44.3)	48 (50.5)	30 (37.5)	0.084(*)
Heparin dose				
Prophylactic dose	95 (55.6)	45 (48.4)	50 (64.1)	Not suitable for testing
Intermediate dose	58 (33.9)	39 (41.9)	19 (24.4)		
Therapeutic dose	18 (10.5)	9 (9.7)	9 (11.5)		

(*)p-value of Pearson's chi-squared test of independence.

**Figura 3:**
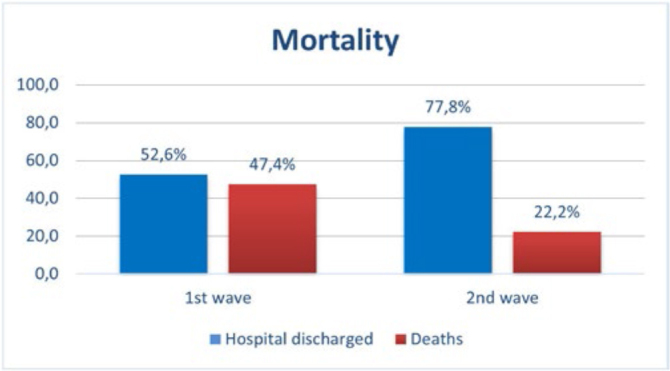
Hospital discharge and death percentage between the 1st and 2nd waves of severe COVID-19 in patients hospitalized in an intensive care unit of a university hospital – Recife, PE, Brazil, 2020-2021.

Mortality was more than double in the 1st wave compared to the 2nd wave (p = 0.001, Figure 3).

Regarding vaccination status, no patient was vaccinated in the first wave and only 7 (8.6%) patients were fully vaccinated for COVID-19 in the second wave.

## DISCUSSION

This study provides a comparative presentation of the evolution of patients with ARDS admitted to the ICU of a university hospital in northeastern Brazil during the 1st and 2nd waves of the COVID-19 pandemic. In the second wave, the number of deaths was reduced by more than half. There was also a tendency for patients to have fewer comorbidities and to develop fewer organ dysfunctions during hospitalization. There were also fewer indications for orotracheal intubation and hemodialysis, but more NIV and corticosteroid therapy.

The evolution of the disease in several countries that became the epicenter of COVID-19 was more severe in the first wave of the pandemic, with higher mortality in older adults, in males, and in those with a greater number of comorbidities^(17,18)^. However, these data varied in the literature when compared between pandemic waves. Younger patients were found in the first wave in Saudi Arabia (47.5 vs. 55, p < 0.001)^(8)^. In a retrospective Brazilian study, there were no significant differences in the mean age (59 years) and percentage of male patients (approximately 55%) among patients hospitalized with ARDS during the first two waves of the pandemic in the Brazilian population, as shown in our study^(19)^.

The most common comorbidities in patients with ARDS in Reus, Spain, were cardiovascular disease, type 2 diabetes mellitus, and chronic neurological disease, and there was no significant difference in the number of comorbidities between waves^(20)^. In the present study, there was a statistical trend (p = 0.051) toward a greater number of comorbidities (≥2) in the first wave. The most common comorbidity in a comparative analysis of data from public ICUs in Brazil was cardiovascular disease^(19)^. Our study showed a higher prevalence of chronic kidney disease (p = 0.005). The need for hemodialysis was almost 3 times higher in the first wave than in the second wave. The incidence of hemodialysis in patients with severe COVID-19 in private and public ICUs in São Paulo (the most populous city and state in Brazil) was 15.7% in the first wave, and 72.5% of these patients died. It was concluded that the need for hemodialysis and the development of a greater number of organ dysfunctions were independent factors of mortality in severe COVID-19^(21)^.

Regarding the admission vital signs, there was a greater number of patients with HR >100 bpm and SBP < 90 mmHg in the first wave, indicating the greater severity of these patients and perhaps justifying the higher mortality observed compared to the second wave. Most of the patients in the public health service were recently triaged to tertiary hospitals, especially in the first wave, with a delay in the care of these patients, thus progressing organic dysfunctions, increasing mortality^(15)^.

Patients in the second wave developed fewer organ dysfunctions according to the SOFA score, which probably contributed to the reduction in mortality. The admission SOFA score was analyzed in 13,301 patients in private ICUs in Brazil from February to October 2020 and showed a median SOFA of zero and there was no statistical difference in the score over the months^(10)^. It is possible that this low number of organ dysfunctions was due to earlier hospitalization and monitoring in Brazilian private sector units.

ICU length of stay was not statistically significant between waves, but hospital length of stay was longer in the second wave (median 17 days), probably due to the higher number of deaths in the first wave, which reduced the length of hospital stay, as well as the higher number of patients with PICS syndrome requiring rehabilitation, which prolonged hospital stay^(4)^. During this period of the pandemic, an outpatient clinic for post-ICU patients was even established, focusing on the rehabilitation of these patients to reduce the morbidity of PICS syndrome and reduce costs and readmissions^(22)^. The unavailability of home oxygen from the public service also made it impossible to discharge them earlier.

In terms of ventilatory support, it was observed that patients admitted in the first wave used more oxygen and more IMV. A similar situation has been described in Reus, Spain^(20)^. However, there was no statistical difference in the use of IMV between waves in Saudi Arabia^(8)^. There was a greater need for oxygen support (11.2% vs. 88.8%) and IMV in the second wave in India (0.9% vs. 24.5%)^(9)^. As observed in our study, the use of invasive ventilation is associated with progression of SARS and increased in-hospital mortality. A similar pattern to our study has been described in Italy, Spain, Japan and Saudi Arabia, with lower mortality and less need for IMV in the second wave^(8,23, 24, 25)^.

The delay in initiating invasive support has also been a well-discussed point in the literature, as the late initiation of support in ARDS could have caused self-inflicted injury leading to inflammation, fibrosis, and irreversible lung damage^(26)^. A worsening respiratory pattern appears to be a marker of increased mortality^(27)^. The delayed arrival of these patients to tertiary centers in the first wave may have had an impact.

The use of corticosteroids in severe COVID-19 has been well established since the publication of the Recovery group study in July 2020, which found that hospitalized patients on oxygen therapy who received 6 mg of dexamethasone for 10 days had lower 28-day mortality and less need for IMV ([RR] 0.83, 95%CI 0.75–0.93)^(28)^. Although the cumulative dose of corticosteroids during hospitalization did not have a significant difference between the waves in the present study, there was an earlier use of this medication in the second wave due to the publication of the Recovery study, and this may have influenced the longer survival, as it must have minimized the disease progression along with less need for IMV compared to the first wave group.

In our reality, as well as worldwide, NIV was not routinely recommended during the first wave due to the high risk of contamination of the team by aerosols and the lack of knowledge about the evolution of the disease^(29)^. An increase in the use of NIV was observed in several private Brazilian ICUs over the months of 2020. This practice may have had an impact on the reduction of mortality over time^(10)^.

There was a general increase in hospital demand and mortality in the second wave in Brazil^(24)^. The VOC gamma variant that emerged in late 2020 in northern Brazil showed greater transmissibility and higher mortality than the alpha variant^(7,30)^.

In addition to viral mutability and host-specific variations, infrastructure and patient care factors need to be evaluated. The ICU of the present study had to be structured more quickly in the first wave to increase the number of beds and to use human resources from different areas. In contrast, the second wave used the already established structure of the ICU and the well-trained team of intensivists. This must have had a direct impact on the reduction in mortality in the second wave. It is already known that inequitable access to beds, major differences between public and private health policies, heterogeneous screening policies, lack of human resources, and lack of adherence to best practices in the management of critically ill patients lead to increased mortality from sepsis and acute respiratory distress syndrome in developing countries^(29)^.

A significant finding of this study was that mortality was 2.1 times higher in the first wave. The mortality in the 1st wave (47.4%) is close to the figures described in Brazilian public ICUs, where the average mortality of patients admitted to ICUs was 55 to 60.5%, while the data from the 2nd wave (22.2%) are similar to those from a private ICU^(11,19)^. A study of private ICUs in Brazil found a 14% mortality rate in 2020, while another Brazilian cohort from a private hospital ICU in São Paulo found a similar average mortality rate^(10,12)^. These studies suggest that this large difference in mortality is related to earlier hospitalization, monitoring, and good hospital practices. In a cross-sectional and comparative study of epidemiological data between the two Brazilian waves in public hospitals, about 66% of patients were admitted to the ICU on the same day of hospital admission, demonstrating how late hospitalization occurred, and it is also observed that there is an inversely proportional relationship between mortality and educational level in COVID-19 in both waves^(19)^.

It is noteworthy that the number of vaccinated patients was still very low in the second wave of the present study. The impact of vaccination in reducing mortality was not achieved in all regions of Brazil until the third wave, even though vaccination started in the second wave^(31)^. This was probably due to late access to vaccination.

This study has several limitations. It is a single-center study, but it is possible to make better comparisons when using the same center, which reduces confounding factors. We had a small number of patients, but there was almost no loss of data or follow-up, so it was possible to compare the clinical-therapeutic behavior of virtually all patients hospitalized in the two periods determined. It was not possible to analyze laboratory data, initial symptoms of the disease and there was no collection of the isolated genotype of SARS-COV-2. Finally, because it is not a randomized study, it is not possible to draw direct conclusions about the impact of clinical-therapeutic interventions.

## CONCLUSION

This is the only work with a comparative data analysis between the first two waves in critically ill patients with severe COVID-19 in Northeast Brazil. To the best of our knowledge, there are no comparative studies that take into account these aspects. In this series, patients with ARDS had lower mortality and fewer comorbidities, less organ dysfunction and less need for IMV and hemodialysis, with greater use of NIV and corticosteroids in the second wave of the pandemic.

ARDS is a syndrome with a high mortality rate, and much of the knowledge gained in the management of patients with severe COVID-19 between the first and second waves allowed an improvement in the management of this pathology. Protocols and hospital flows were created for hospitalization and earlier use of corticosteroids, increasing the chances of survival associated with ARDS. At the same time, the post-ICU outpatient clinic was created to minimize the sequelae of this disease and its treatments.

## DATA AVAILABILITY

All the dataset that supports the results of this study has been published in the article itself.
