# Supplementary material for: Clinical management and outcomes in severe COVID-19: acute respiratory distress syndrome across two waves
Source: Rev Esc Enferm USP. 2025 Jun 20;59:e20240213. doi: 10.1590/1980-220X-REEUSP-2024-0213en (PMC12184099; doi:10.1590/1980-220X-REEUSP-2024-0213en)
Supplement: Translation

# Translation: Gestión clínica y resultados en casos graves de COVID-19: síndrome de distrés respiratorio agudo en dos olas

**DOI:** 10.1590/1980-220X-REEUSP-2024-0213es

**Authors:** Vanessa Cláudia Souza Borba, Simone Cristina Soares Brandão, Lúcia Helena de Oliveira Cordeiro, Michele Maria Gonçalves de Godoy, Maria Cristina Falcão Raposo, Romero Carvalho Coimbra Albêlo, Marina Gabinio de Araújo Pontes, Esdras Marques Lins, Emmanuelle Tenório Albuquerque Madruga Godoy

**Affiliations:** 1 Universidade Federal de Pernambuco, Faculdade de Medicina, Recife, PE, Brasil.; 2 Universidade Federal de Pernambuco, Departamento de Clínica Médica, Recife, PE, Brasil.; 3 Universidade Federal de Pernambuco, Programa de Pós-Graduação em Cirurgia, Recife, PE, Brasil.; 4 Universidade Federal de Pernambuco, Departamento de Estatística, Recife, PE, Brasil.

**Keywords:** Síndrome de Dificultad Respiratoria, Respiración Artificial, Resultados de Cuidados Críticos, Corticoesteroides, COVID-19.

## Abstract

**Objetivo:**

Analizar los cambios en los factores epidemiológicos y en los pronósticos, el manejo clínico y el impacto evolutivo de estas variables en los resultados hospitalarios, comparando las dos primeras oleadas de Síndrome de Distrés Respiratorio Agudo (SDRA) por COVID-19 en un centro universitario del nordeste de Brasil.

**Métodos:**

Se trata de un estudio prospectivo en el que se analizó el perfil clínico, los resultados y el tratamiento de los pacientes hospitalizados. Se incluyeron en la muestra de la primera ola los pacientes hospitalizados desde abril de 2020 a febrero de 2021; la segunda ola, transcurrida entre marzo y agosto de 2021, estuvo asociada al aumento y la disminución de los casos en Pernambuco.

**Resultados:**

De 176 pacientes, 95 pertenecían a la primera oleada y 81, a la segunda. La mortalidad fue del 35,8% y del 47,4% frente al 22,2% (p = 0,001), respectivamente. La edad promedio fue de 55 años [IQR: 46–58], sin diferencias entre las olas. La Evaluación Secuencial del Fallo Orgánico (SOFA) fue mayor en la primera ola, con mediana de 4 [IQR: 3;7,7] frente a 3 [IQR: 2;5,5] y 5 [IQR: 3;8] frente a 3 [IQR: 2;7], en 24 y 72 horas, respectivamente (p = 0,001). Los pacientes de la primera ola recibieron más ventilación mecánica invasiva (VMI), 68,4% frente a 45,7% (p = 0,002) y hemodiálisis, 49,5% frente a 17,7% (p = 0,000), pero menos ventilación no invasiva (VNI), 8,4% frente a 72,5% (p = 0,000) y corticoides, 86,6% frente a 96,6% (p = 0,02). No se vacunó a nadie en la primera ola, mientras que sólo 7 pacientes fueron vacunados en la segunda.

**Conclusiones:**

Los pacientes con SDRA presentaron menos mortalidad, menos disfunciones orgánicas y menos necesidad de VMI y hemodiálisis, con más uso de VNI y corticoides en la segunda ola.

## INTRODUCCIÓN

La fase aguda del síndrome de dificultad respiratoria aguda (SDRA) se caracteriza por la aparición repentina de insuficiencia respiratoria refractaria a la administración de oxígeno a través de un catéter de oxígeno, una mascarilla *no-rebreather* y/o ventilación no invasiva (VNI), que puede progresar hasta la necesidad de ventilación mecánica invasiva (VMI). En particular, el deterioro después de la primera semana de tratamiento adecuado indica un peor pronóstico. En un metaanálisis de 25 estudios que incluyeron a 4881 pacientes con enfermedad por coronavirus 2019 (COVID-19) grave y COVID-19 no grave, la prevalencia de SDRA, disfunción renal y shock fue más común en la COVID-19 grave y la mortalidad de la COVID-19 grave fue de alrededor del 30%^(1)^.

Si un paciente con SDRA desarrolla la necesidad de ventilación mecánica, el monitoreo respiratorio, el manejo de la sedación y el uso de agentes bloqueadores neuromusculares son extremadamente importantes, además de las intervenciones terapéuticas tempranas como la maniobra de reclutamiento alveolar y la maniobra prona^(2)^. La prevención de la lesión pulmonar inducida por el ventilador es uno de los mayores desafíos en el manejo del SDRA debido a los diversos fenotipos de la enfermedad. Es esencial comprender cómo se realizaron estas intervenciones respiratorias^(3)^.

La gravedad de la enfermedad y los tratamientos tienen un impacto directo en las secuelas tras la fase aguda del SDRA. Varios grupos que han estudiado la calidad de vida, la funcionalidad y la mortalidad a largo plazo tras el SDRA han observado varias secuelas físicas, cognitivas y emocionales que se engloban en el síndrome post-cuidados intensivos^(4)^.

Brasil es el tercer país más afectado del mundo por la COVID-19, con aproximadamente 36 millones de casos y casi 700 000 muertes^(5)^. Pernambuco es el séptimo estado más poblado y el décimo más afectado de Brasil por la COVID-19^(6)^. Tras el diagnóstico a gran escala del síndrome respiratorio agudo severo coronavirus 2 (SARS-CoV-2) en varios países, se observó un complejo escenario de oleadas de infección debido a la mutabilidad viral, la relajación de las intervenciones no farmacéuticas, combinado con el bajo nivel de inmunidad adquirida^(7)^. Los factores relacionados con el virus, el huésped y las intervenciones farmacológicas y no farmacológicas han cambiado con el tiempo. Además, no está claro en qué medida esto ha afectado a los resultados hospitalarios ni en qué medida la mejora en el tratamiento clínico de los pacientes ha contribuido a la reducción de la mortalidad.

Es esencial conocer la evolución y el comportamiento del SDRA debido a la COVID-19 y qué factores o intervenciones pueden haber influido positiva o negativamente en los resultados hospitalarios en cada región del mundo para desarrollar un plan de atención y prevención más eficaz en caso de una nueva ola de la enfermedad. El comportamiento de la ola ha sido bastante variable en todo el mundo. En un estudio realizado en Arabia Saudí, se observó una reducción de los síntomas y de la mortalidad en la segunda ola, mientras que en la India la mortalidad fue mayor en la segunda ola^(8,9)^. Existen varios estudios retrospectivos en Brasil que describen el perfil epidemiológico y clínico y los resultados de una sola ola, pero no hay ningún estudio prospectivo que analice comparativamente las olas, teniendo en cuenta las intervenciones terapéuticas y su impacto en la evolución de la enfermedad en Brasil^(10–12)^. Por lo tanto, el objetivo de este estudio fue analizar los cambios en los factores epidemiológicos y pronósticos, el manejo clínico y el impacto evolutivo de estas variables en los resultados hospitalarios, comparando las dos primeras olas en un centro universitario de la región Noreste de Brasil.

Todas estas lecciones aprendidas de la pandemia de COVID-19, especialmente entre la primera y la segunda ola, tienen un impacto positivo en la forma en que se maneja a los pacientes con SDRA para optimizar el tratamiento y reducir la mortalidad y la morbilidad.

## MÉTODO

### Diseño del Estudio y Población

Se trata de un estudio prospectivo y observacional. Se reclutaron 241 pacientes consecutivos mayores de 18 años, ingresados en la Unidad de Cuidados Intensivos (UCI) del Hospital das Clínicas de la Universidad Federal de Pernambuco (EBSERH/HC-UFPE) con una enfermedad respiratoria.

Los pacientes hospitalizados desde el 18 de abril de 2020 hasta el 28 de febrero de 2021 se incluyeron en la muestra de la primera ola; mientras que la segunda ola incluyó a pacientes desde el 1 de marzo hasta el 6 de agosto de 2021, de acuerdo con el aumento y la disminución de casos en Pernambuco catalogados en los boletines epidemiológicos proporcionados por el Centro de Información Estratégica sobre Vigilancia Sanitaria en Pernambuco^(6)^. Se excluyó a 65 pacientes de un total de 241; 55 debido a una prueba de reacción en cadena de la polimerasa con transcripción inversa (RT-PCR) en tiempo real de COVID-19 negativa mediante un hisopo nasal, seis debido a la falta de prueba RT-PCR, dos pacientes con enfermedad terminal avanzada en cuidados paliativos y dos pacientes debido a datos insuficientes en el historial médico. Nuestra muestra consistió en 176 pacientes, 95 en el período de la primera ola y 81 en el período de la segunda ola.

### Recopilación de Datos

Se recopilaron datos demográficos (edad en años y sexo: masculino/femenino), duración de la estancia en la UCI y en el hospital en días, signos vitales al ingreso [frecuencia cardíaca (FC en latidos por minuto - lpm), frecuencia respiratoria (FR en respiraciones por minuto - rpm), presión arterial sistólica (PAS) y presión arterial media (PAM) en mmHg], tipo y número de comorbilidades (obesidad, hipertensión, diabetes mellitus tipo 2, enfermedad pulmonar obstructiva crónica, asma, otras enfermedades pulmonares, enfermedad renal crónica, dislipidemia, enfermedad cardíaca previa), índice de masa corporal (IMC en kg/m^2^ y clasificación de obesidad según el IMC)^(13)^, estado de vacunación contra la COVID-19, intervenciones hospitalarias (uso y modo de administración de oxígeno, uso de ventilación no invasiva (VNI), uso y número de días de ventilación mecánica invasiva (VMI), traqueotomía, uso de fármacos vasoactivos, necesidad de hemodiálisis, realización de maniobra prona (sí o no) o maniobra de reclutamiento alveolar (sí o no), uso de corticosteroides (sí o no), uso de inmunosupresores (sí o no)), uso de corticosteroides (tipo y dosis acumulada en equivalentes de miligramos de dexametasona), uso de heparina (tipo y dosis utilizada durante los primeros siete días después del ingreso), uso de otros medicamentos (sedantes, agentes bloqueadores neuromusculares, oseltamivir, ivermectina y antibióticos) y mortalidad. Las puntuaciones pronósticas del Simplified Acute Physiology Score III (SAPS 3) se calcularon al ingreso, y las puntuaciones de la Sequential Organ Failure Assessment (SOFA) se calcularon al ingreso (24 horas) y 72 horas después del ingreso en la UCI para medir la disfunción orgánica (renal, neurológica, pulmonar, circulatoria, hepática o de la coagulación)^(14,15)^.

Para los pacientes en estado crítico que no podían ser pesados y medidos por métodos tradicionales, la altura y el peso se estimaron utilizando la fórmula de Chumlea, que tiene en cuenta el sexo, la altura de la rodilla, la circunferencia de la pantorrilla y del brazo y el tamaño del pliegue cutáneo subescapular^(16)^. Todas las variables anteriores se recogieron de los registros médicos y se actualizaron diariamente.

El protocolo institucional definió la dosis de heparina como una dosis profiláctica para prevenir la tromboembolia venosa de 40 mg de enoxaparina al día o 5000 UI de heparina no fraccionada de 2 a 3 veces al día, ambas administradas por vía subcutánea. La dosis terapéutica para la tromboembolia venosa es de 1 mg/kg de enoxaparina cada 12 horas o 250 UI/kg de heparina no fraccionada cada 12 horas, mientras que la dosis intermedia sería una dosis entre estas dos.

### Aspectos Éticos

Este proyecto fue aprobado por el Comité de Ética en Investigación del Hospital das Clínicas de la Universidad Federal de Pernambuco, mediante el Dictamen n.º 4,165,300, el 21 de julio de 2020. Los participantes en el estudio autorizaron su participación firmando el formulario de consentimiento informado, o sus familiares lo autorizaron a distancia si no podían hacerlo por razones clínicas.

### Análisis Estadístico

Se utilizó un análisis estadístico descriptivo, que incluyó el cálculo de medias aritméticas, medianas, desviaciones estándar y percentiles para variables cuantitativas (discretas o continuas) y análisis de frecuencia y porcentaje para datos categóricos.

Se utilizó la prueba de Mann-Whitney para comparar medias/medianas, y la prueba de chi-cuadrado de Pearson para examinar asociaciones.

Se realizaron análisis comparativos entre las dos fases utilizando posibles variables explicativas con un nivel de significación (valor p) inferior a 0,05, Los análisis estadísticos se realizaron utilizando el programa SPSS Statistics para Windows, versión 20.0.

## RESULTADOS

Un total de 98 de los 176 pacientes seleccionados eran hombres (55,7%) (p = 0,235). La edad media fue de 55,5 años [Rango inter cuartílico RIC] [46;58], sin diferencias estadísticas en cuanto a sexo y edad entre las dos oleadas (p = 0,10). La duración de la estadía en la UCI fue similar en ambas oleadas, con una mediana de 7 días en ambos RIC (3;18) frente a (3;13,5) para la primera y segunda oleadas, respectivamente (p = 0,335). Sin embargo, la duración de la estadía en el hospital fue mayor en la segunda ola, 12 RIC [6; 27] frente a 17 días [10; 32,5] (p = 0,037).

La mediana del número de comorbilidades no fue estadísticamente diferente entre la primera y la segunda ola (p = 0,140), pero hubo una tendencia hacia un mayor número de comorbilidades (≥2 comorbilidades) en la primera ola, siendo la diabetes la más común. El número de pacientes con enfermedad renal crónica (*clearance* de creatinina <30 ml/min) fue estadísticamente mayor en la primera ola (Tabla 1).

**Tabla 1:** Caracterización de las comorbilidades clínicas en pacientes del estudio hospitalizados con COVID-19 grave en una unidad de cuidados intensivos de un hospital universitario durante la 1ª y la 2ª ola de la pandemia – Recife, PE, Brasil, 2020-2021.

Comorbilidades	Muestra total N = 176 n (%)	1a Ola N = 95 n (%)	2^n^ Ola N = 81 n (%)	p-valor
Obesidad (IMC > 30 kg/m_2_)	78 (48,8)	34 (35,8)	44 (54,3)	0,153(*)
Clasificación nutricional			
<30	82 (51,3)	45 (57,0)	37 (45,7)	0,507(*)
30–35	44 (27,5)	18 (22,8)	26 (32,1)	
35–40	19 (11,9)	9 (11,4)	10 (12,3)	
>40	15 (9,4)	7 (8,9)	8 (9,9)	
Hipertensión	99 (56,3)	53 (55,8)	46 (56,8)	0,894(*)
Diabetes mellitusTipo 2	51 (29,0)	33 (34,7)	18 (22,2)	0,068(*)
Enfermedad pulmonar obstructiva crónica	7 (4)	4 (4,2)	3 (3,7)	0,864(*)
Asma	10 (5,7)	3 (3,2)	7 (8,6)	0,117(*)
Otras enfermedades pulmonares crónicas	5 (2,8)	4 (4,2)	1 (1,2)	0,236(*)
Enfermedad renal crónica	25(14,2)	20 (21,1)	5 (6,2)	0,005(*)
Dislipidemia	3 (1,7)	2 (2,1)	1 (1,2)	0,656(*)
Enfermedad cardíaca previa	24 (13,6)	15 (15,8)	9 (11,1)	0,367(*)
Cantidad de comorbilidades			
0	55 (31,3)	29 (30,5)	26 (32,1)	
1	53 (30,1)	23 (24,2)	30 (37,0)	
2	40 (22,7)	24 (25,3)	16 (19,8)	
3	21 (11,9)	13 (13,7)	8 (9,9)	
4	7 (4,0)	6(6,3)	1 (1,2)	
Mediana (P_25_; P_75_)	1,0 (0,0; 2,0)	1,0(0,0; 2,0)	1,0 (0,0; 2,0)	0,140(**)
Cantidad de comorbilidades			
Hasta 1	108 (66,4)	52 (54,7)	56 (69,1)	0,051(*)
≥2	68 (36,6)	43 (45,3)	25 (30,9)	

(*) Valor p de la prueba de independencia de la Chi-cuadrado de Pearson;

(**) Valor p de la prueba de Mann-Whitney. Abreviatura: IMC = Índice de Masa Corporal.

En la primera ola, 9 (5%) pacientes tenían PAS <90 mmHg al ingreso, frente a 1 (1,2%) en la segunda ola (p = 0,03). La FC media también fue estadísticamente mayor en la primera ola (99 lpm × 92 lpm, 1ª vs. 2,ª ola, p = 0,021). No hubo diferencias en el número de pacientes con PAM < 65 mmHg (0 frente a 5 puntos, 1ª frente a 2ª ola, p = 0,136) y en la frecuencia respiratoria media (29 lpm frente a 26 lpm, 1ª frente a 2ª ola, p = 0,284). Las puntuaciones SOFA a las 24 y 72 horas fueron más bajas en la segunda ola (p = 0,001) (Figura 1). Sin embargo, la puntuación SAPS3 no mostró una diferencia estadística (mediana 14,5 (8;34,5) en la primera ola frente a 15,9 (7,2;20,5) en la segunda ola, p = 0,326).

**Figura 1:** Análisis comparativo de las puntuaciones SOFA de 24 y 72 horas entre la primera y la segunda ola de COVID-19 en un hospital universitario público – Recife, PE, Brasil, 2020-2021, *Valor p de la prueba de Mann-Whitney.

Abreviatura: SOFA = Sequential Organ Failure Assessment (Evaluación secuencial de insuficiencia orgánica).

Se utilizó más oxígeno en forma de catéter de oxígeno (63,2% frente a 40%, p = 0,002) y mascarilla *no rebreather* (94,7% frente a 41, 3%, p = 0,000) en la ola 1, ya que también se colocó a más pacientes en VMI (68,4% frente a 45,7%, p = 0,002) y se sometió a hemodiálisis (49,5% frente a 17,7%, p = 0,000). Sin embargo, menos pacientes recibieron corticosteroides (86,3% frente a 96,3%, p = 0,022) y VNI (8,4% frente a 72,5%, p = 0,000) en la ola 1 (Figura 2).

**Figura 2:** Análisis comparativo del porcentaje de pacientes que requirieron ventilación mecánica invasiva, ventilación no invasiva, corticosteroides y hemodiálisis entre la primera y la segunda ola de COVID-19 grave en un hospital público universitario (Recife, PE, Brasil, 2020-2021).

Abreviatura: ventilación mecánica invasiva (VMI), ventilación no invasiva (VNI).

La mediana de días con ventilación mecánica fue de 11,5 [RIC] [7;19] y no hubo diferencias estadísticamente significativas entre las oleadas. Aproximadamente el 13,7% (n = 13) de los pacientes tuvieron una traqueotomía en la primera oleada y el 20% (n = 16) en la segunda oleada, pero sin significación estadística (p = 0,263). El uso de medicamentos vasoactivos fue similar entre las oleadas (45,3% frente a 42,5%; p = 0,714), al igual que el porcentaje de pacientes sometidos a reclutamiento alveolar y maniobras prono (52,6% frente a 55% (p = 0,754); y 72,6% frente a 65,6% (p = 0,12), respectivamente). La dosis equivalente acumulada mediana de dexametasona fue de 100 mg [RIC] [13;175] y no hubo diferencias estadísticas entre las oleadas (p = 0,178). Hubo un mayor uso de oseltamivir, ivermectina y sedación en la primera oleada (Tabla 2).

**Tabla 2:** Caracterización del uso de medicamentos en pacientes del estudio hospitalizados con COVID-19 grave en la unidad de cuidados intensivos de un Hospital Universitario durante la 1ª y 2ª olas de la pandemia – Recife, PE, Brasil, 2020-2021.

Variable	Muestra total N = 176 n (%)	1ª ola N = 95 n (%)	2ª ola N = 81 n (%)	p-valor
Medicamentos				
Antibióticos	169 (96)	93 (97,9)	76 (93,8)	0,169(*)
Oseltamivir	55 (31,3)	54 (56,8)	1 (1,3)	0,000(*)
Ivermectina	51 (29)	43 (45,3)	8 (10)	0,000(*)
Sedación	95 (54)	60 (63,2)	35 (43,8)	0,010(*)
Bloqueante neuromuscular	78 (44,3)	48 (50,5)	30 (37,5)	0,084(*)
Dosis de heparina				
Dosis profiláctica	95 (55,6)	45 (48,4)	50 (64,1)	No apto para pruebas
Dosis intermedia	58 (33,9)	39 (41,9)	19 (24,4)	
Dosis terapéutica	18 (10,5)	9 (9,7)	9 (11,5)	

(*) Valor p de la prueba de independencia chi-cuadrado de Pearson.

La mortalidad fue más del doble en la primera ola en comparación con la segunda ola (p = 0,001, Figura 3).

**Figura 3:** Porcentaje de altas hospitalarias y muertes entre la primera y la segunda ola de COVID-19 grave en pacientes hospitalizados en una unidad de cuidados intensivos de un hospital universitario – Recife, PE, Brasil, 2020-2021.

En cuanto al estado de vacunación, ningún paciente fue vacunado en la primera ola y solo 7 (8,6%) pacientes fueron completamente vacunados contra la COVID-19 en la segunda ola.

## DISCUSIÓN

Este estudio ofrece una presentación comparativa de la evolución de los pacientes con SDRA ingresados en la UCI de un hospital universitario del noreste de Brasil durante la primera y la segunda ola de la pandemia de COVID-19, En la segunda ola, el número de muertes se redujo en más de la mitad. También se observó una tendencia a que los pacientes tuvieran menos comorbilidades y desarrollaran menos disfunciones orgánicas durante la hospitalización. También hubo menos indicaciones de intubación orotraqueal y hemodiálisis, pero más de VNI y terapia con corticosteroides.

La evolución de la enfermedad en varios países que se convirtieron en el epicentro de la COVID-19 fue más grave en la primera ola de la pandemia, con una mayor mortalidad en adultos mayores, hombres y personas con un mayor número de comorbilidades^(17,18)^. Sin embargo, estos datos variaron en la literatura cuando se compararon entre las olas pandémicas. En la primera ola se encontraron pacientes más jóvenes en Arabia Saudí (47,5 frente a 55, p < 0,001)^(8)^. En un estudio retrospectivo brasileño, no hubo diferencias significativas en la edad media (59 años) y el porcentaje de pacientes varones (aproximadamente el 55%) entre los pacientes hospitalizados con SDRA durante las dos primeras olas de la pandemia en la población brasileña, como se muestra en nuestro estudio^(19)^.

Las comorbilidades más comunes en pacientes con SDRA en Reus, España, fueron enfermedad cardiovascular, diabetes mellitus tipo 2 y enfermedad neurológica crónica, y no hubo diferencias significativas en el número de comorbilidades entre las oleadas^(20)^. En el presente estudio, hubo una tendencia estadística (p = 0,051) hacia un mayor número de comorbilidades (≥2) en la primera oleada. La comorbilidad más común en un análisis comparativo de datos de UCI públicas en Brasil fue la enfermedad cardiovascular^(19)^. Nuestro estudio mostró una mayor prevalencia de enfermedad renal crónica (p = 0,005). La necesidad de hemodiálisis fue casi 3 veces mayor en la primera ola que en la segunda. La incidencia de hemodiálisis en pacientes con COVID-19 grave en UCI privadas y públicas en São Paulo (la ciudad y el estado más poblados de Brasil) fue del 15,7% en la primera ola, y el 72,5% de estos pacientes fallecieron. Se concluyó que la necesidad de hemodiálisis y el desarrollo de un mayor número de disfunciones orgánicas eran factores independientes de mortalidad en la COVID-19 grave^(21)^.

En cuanto a los signos vitales al ingreso, hubo un mayor número de pacientes con FC > 100 lpm y PAS < 90 mmHg en la primera ola, lo que indica la mayor gravedad de estos pacientes y tal vez justifique la mayor mortalidad observada en comparación con la segunda ola. La mayoría de los pacientes del servicio de salud pública fueron recientemente derivados a hospitales terciarios, especialmente en la primera ola, con un retraso en la atención de estos pacientes, lo que hizo progresar las disfunciones orgánicas y aumentó la mortalidad^(15)^.

Los pacientes de la segunda ola desarrollaron menos disfunciones orgánicas según la puntuación SOFA, lo que probablemente contribuyó a la reducción de la mortalidad. La puntuación SOFA de ingreso se analizó en 13 301 pacientes en UCI privadas de Brasil de febrero a octubre de 2020 y mostró una mediana de SOFA de cero y no hubo diferencias estadísticas en la puntuación a lo largo de los meses^(10)^. Es posible que este bajo número de disfunciones orgánicas se deba a una hospitalización y un monitoreo más tempranos en las unidades del sector privado brasileño.

La duración de la estadía en la UCI no fue estadísticamente significativa entre las olas, pero la duración de la estadía en el hospital fue más larga en la segunda ola (mediana de 17 días), probablemente debido al mayor número de muertes en la primera ola, que redujo la duración de la estadía en el hospital, así como al mayor número de pacientes con síndrome PICS que requirieron rehabilitación, lo que prolongó la estadía en el hospital^(4)^. Durante este periodo de la pandemia, se creó incluso una clínica ambulatoria para pacientes post-UCI, centrada en la rehabilitación de estos pacientes para reducir la morbilidad del síndrome PICS y reducir los costes y los reingresos^(22)^. La falta de disponibilidad de oxígeno domiciliario por parte del servicio público también hizo imposible darles el alta antes.

En cuanto al soporte ventilatorio, se observó que los pacientes ingresados en la primera ola utilizaban más oxígeno y más VMI. Se ha descrito una situación similar en Reus, España^(20)^. Sin embargo, no hubo diferencias estadísticas en el uso de la VMI entre las oleadas en Arabia Saudí^(8)^. Hubo una mayor necesidad de soporte de oxígeno (11,2% frente a 88,8%) y de VMI en la segunda oleada en la India (0,9% frente a 24,5%)^(9)^. Como se observó en nuestro estudio, el uso de ventilación invasiva está asociado con la progresión del SARS y el aumento de la mortalidad hospitalaria. En Italia, España, Japón y Arabia Saudí se ha descrito un patrón similar al de nuestro estudio, con una menor mortalidad y menos necesidad de VMI en la segunda ola^(8,23–25)^.

El retraso en el inicio del soporte invasivo también ha sido un punto muy debatido en la literatura, ya que el inicio tardío del soporte en el SDRA podría haber causado lesiones autoinfligidas que condujeran a inflamación, fibrosis y daño pulmonar irreversible^(26)^. Un patrón respiratorio que empeora parece ser un marcador de aumento de la mortalidad^(27)^. La llegada tardía de estos pacientes a los centros terciarios en la primera ola puede haber tenido un impacto.

El uso de corticosteroides en la COVID-19 grave ha quedado bien establecido desde la publicación del estudio del grupo Recovery en julio de 2020, que descubrió que los pacientes hospitalizados en oxigenoterapia que recibieron 6 mg de dexametasona durante 10 días tuvieron una menor mortalidad a los 28 días y menos necesidad de ventilación mecánica invasiva ([RR] 0,83, IC del 95%: 0,75–0,93)^(28)^. Aunque la dosis acumulada de corticoesteroides durante la hospitalización no mostró una diferencia significativa entre las oleadas en el presente estudio, hubo un uso más temprano de este medicamento en la segunda oleada debido a la publicación del estudio Recovery, y esto puede haber influido en la mayor supervivencia, ya que debe haber minimizado la progresión de la enfermedad junto con una menor necesidad de VMI en comparación con el grupo de la primera ola.

En nuestra realidad, así como en todo el mundo, la VNI no se recomendó de forma rutinaria durante la primera ola debido al alto riesgo de contaminación del equipo por aerosoles y a la falta de conocimiento sobre la evolución de la enfermedad^(29)^. En varias UCI privadas brasileñas se observó un aumento en el uso de la VNI a lo largo de los meses de 2020. Esta práctica puede haber tenido un impacto en la reducción de la mortalidad a lo largo del tiempo^(10)^.

Hubo un aumento general de la demanda hospitalaria y de la mortalidad en la segunda ola en Brasil^(24)^. La variante de interés (VOC) gamma que surgió a finales de 2020 en el norte de Brasil mostró una mayor transmisibilidad y una mayor mortalidad que la variante alfa^(7,30)^. Además de la mutabilidad viral y las variaciones específicas del huésped, es necesario evaluar la infraestructura y los factores de atención al paciente. La UCI del presente estudio tuvo que estructurarse más rápidamente en la primera ola para aumentar el número de camas y utilizar recursos humanos de diferentes áreas. En cambio, en la segunda ola se utilizó la estructura ya establecida de la UCI y el equipo de intensivistas bien capacitado. Esto debe haber tenido un impacto directo en la reducción de la mortalidad en la segunda ola. Ya se sabe que el acceso desigual a las camas, las grandes diferencias entre las políticas sanitarias públicas y privadas, las políticas de cribado heterogéneas, la falta de recursos humanos y la falta de adhesión a las mejores prácticas en el tratamiento de pacientes en estado crítico conducen a un aumento de la mortalidad por sepsis y síndrome de dificultad respiratoria aguda en los países en desarrollo^(29)^.

Un hallazgo significativo de este estudio fue que la mortalidad fue 2,1 veces mayor en la primera ola. La mortalidad en la 1^ª^ ola (47,4%) se acerca a las cifras descritas en las UCI públicas brasileñas, donde la mortalidad media de los pacientes ingresados en las UCI fue del 55 al 60,5%, mientras que los datos de la 2^ª^ ola (22,2%) son similares a los de una UCI privada^(11,19)^. Un estudio de UCI privadas en Brasil encontró una tasa de mortalidad del 14% en 2020, mientras que otra cohorte brasileña de una UCI de un hospital privado en São Paulo encontró una tasa de mortalidad promedio similar^(10,12)^. Estos estudios sugieren que esta gran diferencia en la mortalidad está relacionada con una hospitalización más temprana, el monitoreo y las buenas prácticas hospitalarias. En un estudio transversal y comparativo de datos epidemiológicos entre las dos oleadas brasileñas en hospitales públicos, alrededor del 66% de los pacientes ingresaron en la UCI el mismo día de su ingreso en el hospital, lo que demuestra lo tardío que fue el ingreso hospitalario, y también se observa que existe una relación inversamente proporcional entre la mortalidad y el nivel educativo en la COVID-19 en ambas oleadas^(19)^.

Cabe destacar que el número de pacientes vacunados seguía siendo muy bajo en la segunda ola del presente estudio. El impacto de la vacunación en la reducción de la mortalidad no se logró en todas las regiones de Brasil hasta la tercera ola, a pesar de que la vacunación comenzó en la segunda ola^(31)^. Esto se debió probablemente al acceso tardío a la vacunación.

Este estudio tiene varias limitaciones. Es un estudio unicéntrico, pero es posible hacer mejores comparaciones cuando se utiliza el mismo centro, lo que reduce los factores de confusión. Tuvimos un pequeño número de pacientes, pero casi no hubo pérdida de datos ni de seguimiento, por lo que fue posible comparar el comportamiento clínico-terapéutico de prácticamente todos los pacientes hospitalizados en los dos períodos determinados. No fue posible analizar los datos de laboratorio, los síntomas iniciales de la enfermedad y no se recogió el genotipo aislado del SARS-COV-2, Por último, al no tratarse de un estudio aleatorio, no es posible extraer conclusiones directas sobre el impacto de las intervenciones clínico-terapéuticas.

## CONCLUSIÓN

Este es el único trabajo con un análisis de datos comparativo entre las dos primeras olas en pacientes críticos con COVID-19 grave en el noreste de Brasil. Hasta donde sabemos, no existen estudios comparativos que tengan en cuenta estos aspectos. En esta serie, los pacientes con SDRA tuvieron una menor mortalidad y menos comorbilidades, menos disfunción orgánica y menos necesidad de VMI y hemodiálisis, con un mayor uso de VNI y corticosteroides en la segunda ola de la pandemia. El SDRA es un síndrome con una alta tasa de mortalidad, y gran parte del conocimiento adquirido en el manejo de pacientes con COVID-19 grave entre la primera y la segunda ola permitió una mejora en el manejo de esta patología. Se crearon protocolos y flujos hospitalarios para la hospitalización y el uso más temprano de corticosteroides, aumentando las posibilidades de supervivencia asociadas al SDRA. Al mismo tiempo, se creó la clínica ambulatoria post-UCI para minimizar las secuelas de esta enfermedad y sus tratamientos.

## DISPONIBILIDAD DE DATOS

Todos los conjuntos de datos que respaldan los resultados de este estudio se han publicado en el propio artículo.
